# Female Pattern Hair Loss: An Overview with Focus on the Genetics

**DOI:** 10.3390/genes14071326

**Published:** 2023-06-23

**Authors:** Chih-Yi Ho, Jeff Yi-Fu Chen, Wen-Li Hsu, Sebastian Yu, Wei-Chiao Chen, Szu-Hao Chiu, Hui-Ru Yang, Sheng-Yao Lin, Ching-Ying Wu

**Affiliations:** 1 School of Post Baccalaureate Medicine, College of Medicine, Kaohsiung Medical University, Kaohsiung 807, Taiwan; 2 Department of Biotechnology, Kaohsiung Medical University, Kaohsiung 807, Taiwan; 3 Department of Dermatology, College of Medicine, Kaohsiung Municipal Ta-Tung Hospital, Kaohsiung Medical University Hospital, Kaohsiung Medical University, Kaohsiung 801, Taiwan; 4 Department of Laboratory Medicine, Kaohsiung Municipal Ta-Tung Hospital, Kaohsiung 801, Taiwan; 5 Department of Cosmetic Science, Chang Gung University of Science and Technology, Taoyuan 333, Taiwan

**Keywords:** female pattern hair loss, genetic factors, single nucleotide polymorphism (SNP), androgenetic alopecia, sex hormones, targeted gene therapy

## Abstract

Pattern hair loss can occur in both men and women, and the underlying molecular mechanisms have been continuously studied in recent years. Male androgenetic alopecia (M-AGA), also termed male pattern hair loss, is the most common type of hair loss in men. M-AGA is considered an androgen-dependent trait with a background of genetic predisposition. The interplay between genetic and non-genetic factors leads to the phenotype of follicular miniaturization. Although this similar pattern of phenotypic miniaturization can also be found in female pattern hair loss (FPHL), the corresponding genetic factors in M-AGA do not account for the phenotype in FPHL, indicating that there are different genes contributing to FPHL. Therefore, the role of genetic factors in FPHL is still uncertain. Understanding the genetic mechanism that causes FPHL is crucial for the future development of personalized treatment strategies. This review aims to highlight the differences in the ethnic prevalence and genetic background of FPHL, as well as the current genetic research progress in nutrition, Wnt signaling, and sex hormones related to FPHL.

## 1. Introduction

**Figure 1:**
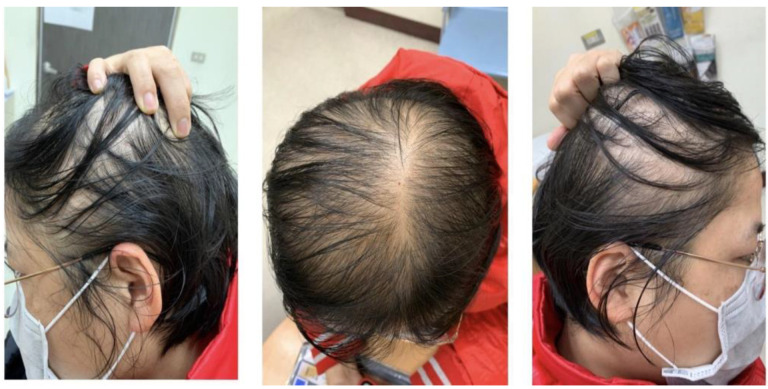
Hair manifestations in women with FPHL.

Female pattern hair loss (FPHL) is commonly seen in women. Although in most cases it is a non-scarring, non-inflammatory condition, loss of hair density often leads to disfigurement, afflicting most patients. The disease is clinically characterized by gradual loss of hair in the central and forehead regions of the scalp, but preservation of the forehead hairline. (Figure 1) This pattern differs from that seen in male androgenetic alopecia (M-AGA), which is often characterized by a receding frontal hairline, followed by baldness and possibly central hair loss. Although the appearance may be different, the final follicular changes in both disorders are similar, such as perifollicular microinflammation, increased number of atrophic follicles, reduced sebaceous gland volume, and reduced anagen-to-telogen ratio [1,2]. Given that these two diseases eventually lead to typical follicular miniaturization and hair loss, it has long been believed that the pathogenesis of female pattern hair loss should be similar to that of M-AGA. It has been recognized that M-AGA is due to a genetically determined androgen-dependent trait; however, the exact genetic cause of FPHL remains unclear, and the direct relationship between androgens and FPHL is yet to be elucidated. So far, accumulating studies have suggested that the pathogenesis of these two diseases is not identical.

**Figure 2:**
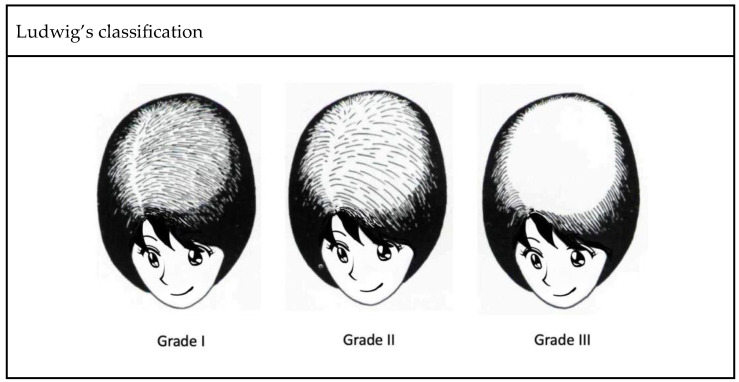

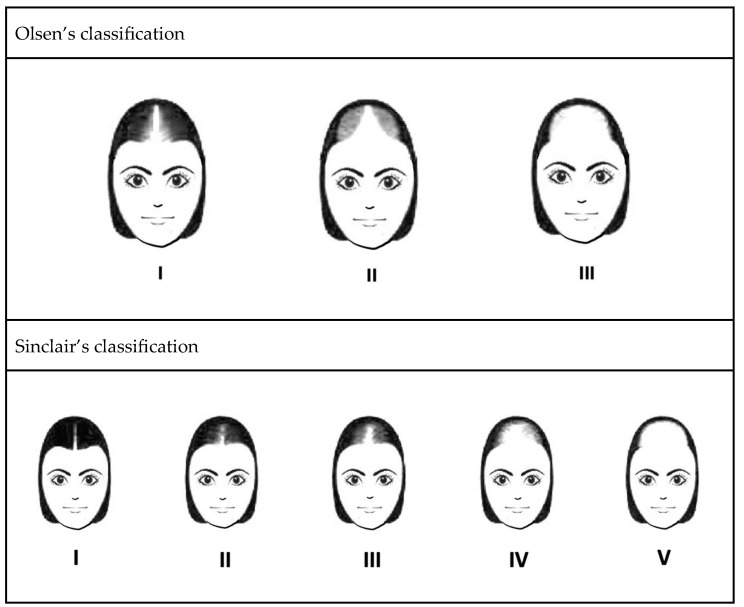
Three well-recognized classification systems of FPHL.

In clinical practice, several classification systems are used to grade the severity of FPHL. Three commonly used grading methods are the Ludwig classification, Olsen classification, and Sinclair classification (Figure 2). First proposed in 1977, the Ludwig classification is a widely recognized system that divides FPHL into three grades, focusing on the central portion of the scalp and the frontal hairline. It provides an intuitive method to assess the severity of hair thinning and helps determine appropriate treatment strategies [3]. Olsen’s classification is based on the density of the hair and the visibility of the scalp. It takes into account preservation of the anterior hairline, vertex involvement, and general degree of hair loss, with emphasis on forehead protrusion [4]. Sinclair’s classification evaluates hair thinning along the midline and further ranks the severity of hair loss into five grades [5]. Compared with previous methods, this grading system provides a finer classification, making it potentially suitable for the early detection of FPHL. These grading systems, including those of Ludwig, Olsen, and Sinclair, are valuable tools for healthcare professionals to assess and classify the severity of FPHL. Using these grading methods, individualized treatment plans can be formulated and the progress of the disease can be monitored effectively.

FPHL is not only a common hair problem, it also profoundly impacts patients’ psychological and social well-being [6]. FPHL could cause severe psychological stress, leading to depression, anxiety, and damaged self-esteem, which may further affect patients’ interpersonal relationships, work, and quality of life [7]. A previous study indicated that FPHL could negatively impact patients’ functioning, emotions, self-confidence, and stigmatization, and some patients may even endure psychological disturbance, such as dysmorphophobia or affective disorder [8]. The article presents several strategies of interventions, such as adopting positive behaviors and attitudes to reduce the negative impact of hair loss. Nonetheless, some patients may experience maladaptive responses, including aimless rumination and anger towards others, which may deteriorate their personal lives, such as marital relationships. Consequently, hair loss and the associated maladaptive responses have a significant impact on the patients. Therefore, understanding FPHL helps in clinical treatment and effectively improves patients’ quality of life. In this review, we describe the epidemiology and etiology of FPHL, focusing on the currently known genetic findings.

## 2. Epidemiology

In 2022, Chaikittisilpa S et al. reported a prevalence of FPHL in 52.2% of 178 postmenopausal women, with a mean age and postmenopausal duration of 58 years and 9 years, respectively [9]. FPHL is a common hair loss disorder, but studies on its prevalence often result in inconsistent conclusions, possibly due to a lack of widely accepted diagnostic criteria to define the disease. Although the incidence rate varies by country, it increases with age, of note, with 55% of women over the age of 70 experiencing significant pattern hair loss [1]. Severe disease progression occurs during adolescence in a small number of patients. Typically, FPHL reaches a first peak during the reproductive years and a second peak after menopause [1]. Based on this observation, it is believed that FPHL is closely related to hormone changes.

Interestingly, the prevalence of FPHL also varies by place of residence and ethnicity. For example, a prevalence analysis of a student population found that the overall prevalence of FPHL among junior high school girls is 28.6%, which is significantly higher in rural areas than in urban areas, and is significantly associated with family history [10]. Although FPHL can occur in all races, the prevalence of Caucasian women was reported to be higher than that of Chinese and Korean women [1] (Table 1).

## 3. Etiology and Pathogenesis

**Figure 3:**
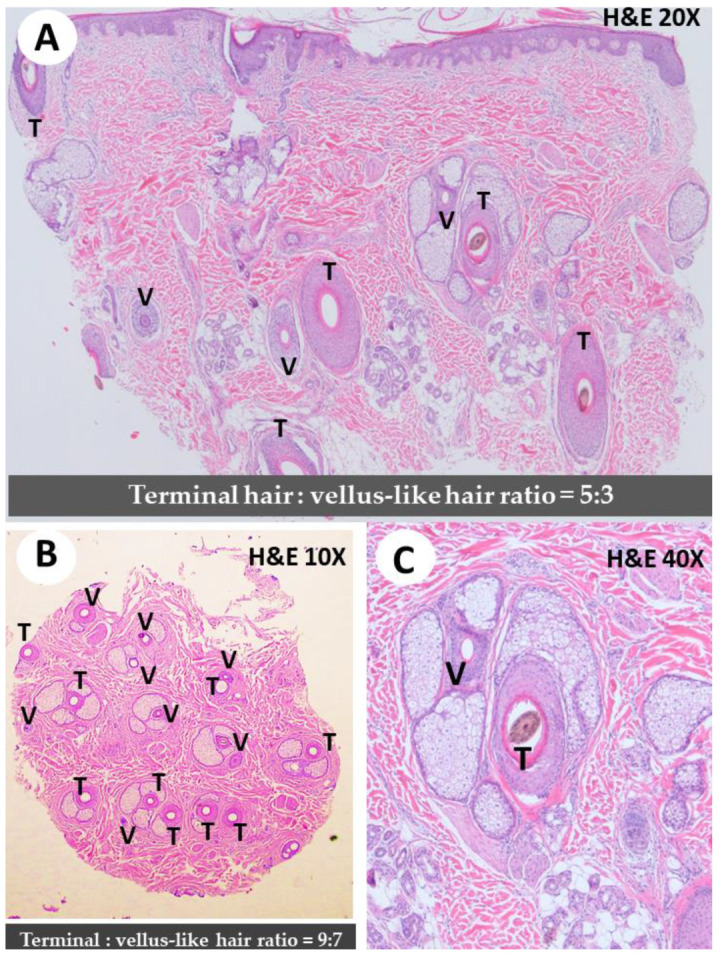
Histological findings in pattern hair loss (M-AGA and FPHL). (**A**) Follicles miniaturization with increased vellus (V) hair follicles versus terminal hair (T) (T/V ratio = 5/3) × 20. (**B**) Diffuse miniaturization with increased vellus (V) hair follicles. (T/V ratio = 9/7). Increased fibrous tissue surrounding the follicles was seen. ×10. (**C**) Note variation in hair follicle size × 40. An increased vellus hair to terminal hair of the affected area is diagnostic of pattern hair loss (M-AGA and FPHL). The normal values of the T:V ratio on the non-alopecia scalp are approximately 2/1 to 7/1 [14].

The pathophysiology of FPHL has not been fully elucidated. Evidence suggests it may involve genetics, sex steroid hormones, and environmental factors [11]. The final pathological manifestations of M-AGA and FPHL are similar, both of which are follicle miniaturization, characterized by an increase in the proportion of vellus hairs and a decrease in the ratio of terminals to vellus. Along this process, a reduction in the size of the sebaceous glands, an increase in the number of microfollicles, and a decrease in the anagen-to-telogen ratio can easily be observed [1,12]. Histopathological features of FPHL include miniaturization of hair follicles, reduced follicle density, and increased fibrous tissue surrounding the follicles (Figure 3). Miniaturization describes the process by which normal terminal hairs (T) become sparse vellus hairs (V). A normal unaffected scalp typically has a T/V ratio of approximately 4/1 to 7/1 [13]. In PHL of both genders, however, the number of vellus (V) and intermediate hair follicles is increased, and the ratio of terminal (T) to vellus (V) hairs is usually less than 2/1 [14]. The affected follicles undergo a shorter growth phase (anagen) and an extended resting phase (telogen). Additionally, inflammation and perifollicular lymphocytic infiltrates may be observed. These pathological changes lead to progressive hair thinning and loss in a pattern typically localized to the central scalp. The exact cause of this degeneration is unknown; however, arrector pili muscle degeneration, inflammation, and other mechanisms have been proposed.

In a recent study investigating the correlation between inflammation and hair loss, Nadia Peyravian et al. [15] identified a role for inflammation in the pathogenesis of hair loss in men and women, with supportive evidence of clinical, histological, and sequencing findings. Perifollicular inflammatory cell infiltration was demonstrated in biopsy specimens from patients with M-AGA and FPHL. Significant infiltration of CD4+ T cells into hair follicles was also shown to be responsible for hair cycle changes, leading to M-AGA and FPHL alopecia [15]. Taken together, these results demonstrate that inflammation is a crucial element of the pathophysiology of M-AGA and FPHL, reinforcing the need to address inflammation in future therapeutic development.

## 4. Genetics of FPHL

### 4.1. Mode of Inheritance and Genome-Wide Association Studies (GWAS)

M-AGA is considered a heritable disease [16]. A study of 572 men by MP Birch et al. showed that men with bald fathers were five times more likely to develop M-AGA than men with non-bald fathers [17]. In a case–control study conducted by Jungyoon Ohn et al. in 2022, the proportion of individuals with a positive family history of pattern hair loss is higher in patients with early onset female pattern hair loss compared to those without the disease, with rates of 71.4% versus 51.0% (*p* = 0.004), respectively. Furthermore, a higher correlation of maternal family history was found in patients with early onset FPHL compared to unaffected women, with rates of 33.3% versus 9.1% (*p* < 0.001), respectively [18]. Although the contribution of the paternal side of family history was more frequently implicated in previous studies, it was not significantly relevant in those studies. Although female hair loss is mainly determined by genetic factors, the detailed regulation of the genetic factors remains to be studied.

Genome-wide association studies (GWAS) are a method for identifying genomic variants that are statistically associated with disease risk or specific traits. Genome-wide linkage or association studies of FPHL have not been evaluated and reported in detail, and current studies mainly focus on M-AGA susceptibility loci and genes of sex steroid-related hormone pathways.

### 4.2. Correlation between Nutrition and FPHL

Despite the fact that data supporting the relationship between hair follicle turnover and nutritional deficiencies in FPHL patients are still lacking, many aspects of FPHL treatment are related to nutritional supplementation. A recent study by Piccini [19] clarified that affected intermediate/miniaturized hair follicles in FPHL display relative nutritional deficiency and dormant metabolism, but the function of absorbing nutrients is not impaired, thus suggesting the potential of nutritional supplementation as an adjunctive therapy for FPHL.

The effects of vitamin D on FPHL are still controversial. Vitamin D receptors (VDR) are known to be expressed in follicular keratinocytes and dermal papilla cells and play a role in hair growth regulation. Marwa M T Fawzi et al. [20] demonstrated a crucial role of VDR in the pathogenesis of alopecia areata and AGA by reporting lower serum and tissue VDR levels in alopecia areata and AGA patients compared to the control group. Recently, Iman Seleit et al. [21] conducted a case–control study identifying VDR gene types on 30 FPHL patients with 30 age-matched healthy female subjects as the control group and found that Taq-1 and Cdx-1 from VDR genes could be considered risk factors for FPHL. The genotypes of CC, TC, and T alleles of Taq-1 are associated with FPHL patients, and the CC genotype increases the risk of disease by 12.6 times. The genotypes of AA, GA, and G allele are also associated with FPHL, and the AA genotype increases the risk of disease by 7.5 times. On the other hand, Banihashemi et al. [22] proposed that the concentration of vitamin D3 in the blood is related to the incidence of FPHL, and the vitamin D3 levels were significantly lower in serum samples with FPHL. There is still a lack of relevant research on whether vitamin D supplementation could promote hair growth in FPHL patients with vitamin D deficiency.

Nutrient deficiencies associated with FPHL have been increasingly implicated in recent studies, in which the nutritional/metabolite profile of hair follicles may vary from one patient to another, likely due to differences in dietary, hormones, and genetic background. A low abundance of TCA cycle products, including citric, malic acid and lactate, from aerobic glycolysis were found in occipital or parietal intermediate hair follicles in some patients, whereas L-glutamine was increased in other patients [19]. These findings show that FPHL women may differ in the metabolism of nutrients compared with women without the disease, but whether this result can be confirmed through genetic correlation studies remains to be seen. At present, studies on the genetic aspects of VDR-associated FPHL are still relatively limited, while the impact of other nutrients on FPHL may deserve further investigation.

### 4.3. Polycystic Ovary Syndrome (PCOS) with FPHL

PCOS is a common cause of infertility in women of reproductive age, and FPHL is known to be associated with hyperandrogenism, especially in PCOS patients [23]. The HSD3B1 gene encodes the 3β-hydroxysteroid dehydrogenase-1 (3βHSD1) enzyme, which catalyzes the conversion of adrenal androgen precursors to the most potent androgen, dihydrotestosterone. Genetic variants of HSD3B1 1245C are known to result in increased androgen expression in peripheral tissues. This finding is supported by a genetic study from Taiwan, in which, among 472 recruited PCOS patients, those who had the HSD3B1 1245C allele were more likely to develop FPHL [24]. PCOS patients with FPHL are not only physically afflicted but suffer profound social and psychological difficulties. However, the exact pathogenic mechanism remains to be studied. It is hopeful that advanced studies on the genetic aspects should facilitate treatment, early screening, and diagnosis of PCOS with FPHL in the future.

### 4.4. Dickkopf WNT Signaling Pathway Inhibitor 1

DKK-1 (Dickkopf WNT signaling pathway inhibitor 1) gene encodes a member of the Dickkopf family of proteins. When mutated or overexpressed, WNT is a proto-oncoprotein that can promote cell proliferation and transformation. A study in 2019 revealed that the expression of DKK-1 was significantly increased in patients with AGA and alopecia areata (AA), suggesting that DKK-1 plays a certain role in these hair-related diseases [25].

Kwack et al. conducted a series of studies on the expression of Dickkopf 1 (DKK-1) induced by Ectodysplasin-A2 (EDA-A2). They used siRNA transfection and DKK-1 ELISA, among other methods, and found that DKK-1 expression was increased in the human balding dermal papilla cells treated with EDA-A2, eventually leading to apoptosis [26].

Furthermore, R-spondin 1 (RSPO1), a secreted Wnt signaling agonist that possesses an antagonizing effect against DKK-1, was found to be beneficial to hair condition. A recent study reported that a conditioner containing 2% watercress extract (WCE) improved hair loss symptoms by increasing hair thickness and density through its ability to inhibit DKK-1 secretion and antagonize DKK-1 through RSPO1 [27].

At present, most studies on DKK-1 focus on patients with AGA and AA, but there is increasing evidence that DKK1 influences hair follicle growth. Thus, more in-depth studies on the involvement of DKK1-related pathways in FPHL should shed more light on the pathogenesis of the disease.

### 4.5. Prediction of Dermal Sheath Cup Cell Therapy

The dermal sheath cup (DSC), a tissue located at the base of the dermal sheath (DS), has been shown to contribute to hair follicle regeneration [28].

Hair follicle shrinkage is well established in the mechanism of hair loss in both men and women, and DSC cells may play a role in treating both diseases. Based on these observations, Tsuboi et al. conducted a clinical study of autologous cell-based therapy, demonstrating that transplantation of DSC cells contributed to increased hair density and hair diameter [29].

However, the outcome of DSC cell transplantation varies among patients, prompting Yoshida et al. [28] to screen for changes in gene expression in DSC cells associated with the treatment outcome. They found that negative treatment response to DSC cells can be predicted by detecting markers such as CALD1, ACTA2, and two smooth muscle cell markers MYOCD and SRF. These findings provide potential markers that can help in preclinical assessments of DSC cells.

### 4.6. Sex Steroid Hormones Gene Polymorphism

It is conceivable that sex hormone-related genes are likely involved in regulating FPHL. For example, similar miniaturizing of terminal hair in M-AGA is caused by the binding of dihydrotestosterone to androgen receptors on susceptible hair follicles, leading to the upregulation of genes accounting for hair loss [30]. It is believed that androgen-secreting adrenal or ovarian tumors can result in hyperandrogenism. Women with polycystic ovary syndrome and ovarian androgen-secreting tumors may develop early-onset FPHL, a common feature of these disorders [23]. However, as reported in 2020, the role of androgen in FPHL remains unclear, as some patients with FPHL have normal androgen concentrations [31,32]. This result suggests that the pathophysiology of the disease has yet to be fully understood.

In the genetic research of FPHL, some studies evaluated specific SNPs of CYP19A1 and ESR2 genes, both of which are related to the transformation of sex hormones [33]. Previous genetic studies have identified associations between FPHL and the ESR2 and CYP19A1 genes. ESR2 encodes estrogen receptor beta, the target receptor for estrogen. Variations in this gene may disrupt estrogen signaling and downstream molecular pathways, leading to disease progression. A study by L. Yip et al. [34] analyzed 484 FPHL patients (Sinclair scale 3–5) and 471 controls (Sinclair scale 0) and found a significant association between FPHL and SNPs rs10137185, rs17101774, and rs2022748. However, S. Redler et al. found discordant results for rs101371856 in German and British populations [35]. CYP19A1 encodes aromatase, an enzyme responsible for converting androgens into estrogens. Genetic variants of CYP19A1 may disrupt the balance between estrogen and androgen levels, resulting in this disorder L. Yeh et al. A GWAS analysis of FPHL and CYP19A1, including 484 Australian FPHL patients (Sinclair grade 3–5) and 471 controls, found a significant association between FPHL and the SNP rs4646 CC genotype [36]. Likewise, S. Redler et al. analyzed the association between CYP19A1 and FPHL in 145 UK patients and 53 German patients. They studied four SNPs (rs4646, rs16964189, rs2470158, and rs28757184) but found no significant association between the two groups [37]. A recent study in a Chinese Han population, including 200 FPHL patients and 200 controls, revealed significant differences in the allele frequency and distribution of CYP19A1 SNPs rs6493497 and rs7176005 associated with FPHL [38]. In 2021, a study in a Polish population analyzed the association between 13 CYP19A1 and 11 ESR2 gene SNPs and FPHL. The study included 117 FPHL patients and 128 healthy controls, but no significant differences were observed [33]. To sum up, previous studies have shown an association between FPHL and CYP19A1 and ESR2. However, differences in race and sample size may have influenced the findings, and further genetic analysis are needed to confirm their relationship.

Follicle miniaturization can be affected by dihydrotestosterone (DHT), and testosterone (T) can be converted into DHT or β-estradiol (E2) through 5α-reductase (5α-R) and aromatase, respectively. Therefore, these two enzymes will determine the concentration of DHT and E2, and their ratio will affect hair volume. A study quantifying aromatase and 5α-R isozyme mRNA levels in the hair plucked from young women with FPHL found 5α-R3 mRNA in human hair, but the increase of 5α-R isozyme in FPHL patients was inconsistent [32]. Such results could explain why patients respond inconsistently to medications and also provide new directions for more specific treatments in the future.

Hormone-related treatments are frequently used in FPHL patients, and E2 supplementation has been shown to increase hair growth in ovariectomized (OVX) mice lacking E2 and exhibiting alopecia similar to FPHL [39]; however, the underlying therapeutic mechanism has yet to be elucidated. A Japanese study showed that genes related to the angiopoietin 2 (ANGPT2) pathway were upregulated in mice given E2, and OVX mice treated with ANGPT2 had higher hair density than the control mice. They concluded that the reversal of hair loss is modulated by the estradiol-ANGPT2 axis [40]. Although considerable progress has been made in the past few years toward a better understanding of hormone-related genes in FPHL, there is still a lack of results with significant correlations. In particular, SNP-related studies on the disease-causing, sex hormone metabolism genes are often inconsistent with the findings in M-AGA.

### 4.7. Inconsistency of Association with M-AGA Genetics

The terminal morphology of FPHL is similar to that of M-AGA, which is caused by the miniaturization of hair follicles. Thus, it is speculated that the results of genetic analyses of the two diseases may be highly correlated. While current genetic research on male hair loss has identified a series of susceptibility genes/loci through GWAS [41,42], whether these loci are correlated with female hair loss is still unknown. A study by Liang et al. [43] analyzed blood samples of FPHL patients from a Chinese Han population. They studied the associations between 22 tag SNPs in M-AGA and 82 FPHL patients via the Sequenom iPlex platform. The results showed no significantly associated variants, suggesting that the 22 SNPs examined in M-AGA may not be associated with FPHL. The genetic studies of associations between candidate loci in M-AGA and FPHL are summarized and presented in Table 2 [12,34,36,37,43,44,45,46,47].

Hair follicle degeneration is common in alopecia disorders. In clinical features of M-AGA, hair loss can be seen in the frontal and vertex regions, whereas the occipital region is usually unaffected. In response to these findings, Liu et al. [48] performed transcriptome analysis and in vitro cell experiments on hair follicles from different regions of M-AGA patients. They found that differential expression of HIF-1 prolyl hydroxylases (EGLN1, EGLN3) and Wnt/β-catenin pathway inhibitors (SERPINF1, SFRP2) was associated with the growth of hair follicles. Still, the expression of these association genes in FPHL remains unknown.

## 5. Therapies of FPHL

The treatment of FPHL includes both topical and systemic therapies, and surgical intervention can also be considered for more severe cases. Here, we summarize and introduce the currently recognized treatment options for FPHL.

### 5.1. Standard Management

There are generally several types of options for topical treatment. Minoxidil, a potent vasodilator, is commonly used topically for FPHL treatment. It is available in 2% or 5% solution and 5% foam formulations in the United States. Although the exact mechanism remains unclear, Minoxidil can enhance perifollicular angiogenesis and has been proven effective for FPHL through meta-analysis of randomized trials [49]. Prostaglandin (PG) analogs have been studied for their potential to promote hair growth. Latanoprost, a PG-F2 analog, was found to increase hair density and pigmentation in a study of M-AGA [50]. Setipiprant (KITH-105) is an oral inhibitor of the PG-D2 receptor (GPR44) that may have the potential to promote hair growth [51]. In addition, platelet-rich plasma (PRP) has been utilized for the treatment of hair loss related to M-AGA, FPHL, and AA. Systematic reviews and meta-analyses have indicated that PRP can increase hair thickness and density in the treatment of FPHL [52,53]. Furthermore, low-level light therapy (LLLT) is a relatively new treatment option for hair loss. A review by Egger et al. found that LLLT is effective for both men and women, with significant improvements in hair density and thickness after treatment [54]. LLLT devices have a good safety profile, although the exact therapeutic mechanism remains unclear. Current research suggests that LLLT may promote hair growth by stimulating the electron transport chain in mitochondria, leading to increased energy production [31].

The following are several options available for systemic treatment. Hormone-related treatments are commonly used for male and female androgenetic alopecia. Finasteride, a type II 5-alpha reductase inhibitor, is effective for male androgenetic alopecia, but its use in female androgenetic alopecia is limited due to potential teratogenic effects. Recent studies evaluated the role of finasteride in the treatment of FPHL in more detail. The results showed that topical finasteride may be used as a new option to reduce adverse systemic side effects [55,56,57]. Whereas dutasteride, a second-generation 5-alpha reductase inhibitor, is more effective than finasteride, further studies are needed to determine its safety and effectiveness in female androgenetic alopecia [58]. Cyproterone acetate is an anti-androgen still without a consensus on dosage for female androgenetic alopecia. Indeed, there is insufficient evidence on whether oral hormone therapy prevents or improves FPHL. Spironolactone is an off-label use for female androgenetic alopecia and has a good safety profile. Clinical studies suggest a daily dose of 100–200 mg, with the best response after 1 year or more of treatment [59]. On the other hand, oral Minoxidil is not a preferred first-line treatment for FPHL due to its higher incidence of side effects compared to topical Minoxidil. In a randomized clinical trial involving 52 FPHL patients, oral Minoxidil (1 mg per day) and Minoxidil 5% solution once a day showed no significant difference in treatment outcomes such as hair density, but side effects were more significant in the oral Minoxidil group (27%) than the topical Minoxidil group (4%) [60]. Currently, a low dose of 0.25 mg is commonly used for FPHL patients who receive oral Minoxidil [61].

Surgical intervention can be considered for patients who are unresponsive to drug therapy. Various surgical techniques, including hair transplantation, hair micropigmentation, mesotherapy with growth factors, microneedling, and bioengineered hair follicle stem cells are available. The cost and duration of treatment vary among these techniques, and patients should be fully informed of their options before undergoing surgery.

### 5.2. Miscellaneous

There are numerous innovative treatments for FPHL. A review by Kriteeka Saini et al. [62] investigated the effect of vitamin D supplementation on hair growth and found that vitamin D plays a role in various signaling pathways for hair follicle growth and differentiation. Other nutritional supplements, such as biotin, caffeine, melatonin, and zinc, have been proposed as potential therapies for hair loss, but the exact therapeutic mechanisms remain unclear [63,64,65,66]. While nutritional supplements are often used off-label or as adjuvant therapy in treating FPHL, a large body of evidence is s\ll needed to support their efficacy.

Botanical preparations have gained increasing attention in recent years. Ibrahim et al. [67] conducted a randomized comparative trial with 60 FPHL patients using pumpkin seed oil or minoxidil 5% foam for three months. Although both groups had similar treatment outcomes, pumpkin seed oil was identified as a new and safe treatment option. Campiche et al. [68] identified the effects of *Leontopodium alpinum* in regulating hair follicle growth, including inhibition of hair loss in vitro and an increase in hair regrowth in vivo. Nakamura et al. [69] discovered that water-soluble peptides from egg yolk, also known as hair growth peptide (HGP), stimulate the production of vascular endothelial growth factor (VEGF) and human dermal papilla cell growth by inducing hypoxia-induced factor (HIF)-1a-mediated transcriptional pathways and VEGF expression. With the known side effects of long-term minoxidil use, alternative hair growth strategies have been evaluated. Finally, a study published in 2023 using asymmetric small interfering RNAs (asiRNAs) to downregulate androgen receptor (AR) expression and the downstream AGA-associated signal has led to the finding that AR gene-targeting cell penetrating asiRNA (cp-asiRNA) is effective in FPHL treatment [70].

## 6. Summary of Genetic Data on FPHL, M-AGA, and Other Non-Scarring Alopecia

From the aforementioned studies, it can be summarized that the clinical manifestations are different between M-AGA and FPHL, despite the similar pathological mechanisms of the two diseases. In genetic research, while most studies focus on the correlation between FPHL and the SNPs of M-AGA, only a few demonstrate a positive correlation. Furthermore, there is inconsistency in the expression of the susceptible genes in different ethnic groups, indicating the heterogeneity of genetic factors for FPHL in different populations. For example, although the genetic loci of AR/EDA2R are more related to M-AGA, their association with FPHL in various ethnic groups is not significant. These results suggest that the susceptible genetic loci of FPHL are likely to be different from those of M-AGA. The results of genetic testing for different types of hair loss diseases correspond to their unique genes and corresponding loci. In common non-scarring hair loss disorders such as pattern alopecia, AA, and chronic TE, different hair loss disorders can be attributed to different genetic etiologies, however, in addition to genetic features, there are distinct clinical features and histopathological clues that can help differentiate one from the other (Table 3).

In short, genetic research plays a crucial role in the field of medicine, including genetic diagnosis, gene therapy, genetic screening, and drug target development. Further investigation of genetics and molecular biology is essential for developing more accurate and personalized diagnostic and treatment strategies. Likewise, understanding the genetic specificity of FPHL should facilitate accurate diagnosis and personalized treatment, thereby maximizing treatment efficacy and minimizing unnecessary side effects for the patients. Moreover, in-depth research on genetic specificity can promote understanding of the pathophysiological mechanisms underlying FPHL, paving a new avenue for more future relevant studies.

## Figures and Tables

**Table 1:** Prevalence of FPHL by age group according to epidemiological studies of different races.

Highly Admixed Population							
Study	Region	Age Groups					
		20–29 *	30–39 *	40–49 *	50–59 *	60–69 *	>70 *
2022, Tsutsui	Brazil	9	22	50	69	-	-
Caucasian							
Study	Region	Age groups					
		20–29 *	30–39 *	40–49 *	50–59 *	60–69 *	>70 *
2005, Gan	Australia	12	17	25	28	41	54
2001, Norwood	USA	3	17	16	23	25	29
2001, Birch	England	3	10	5	14	33	38
Asian							
Study	Region	Age groups					
		20–29 *	30–39 *	40–49 *	50–59 *	60–69 *	>70 *
2012, Su	Taiwan	-	6	10	12	13	15
2010, Wang	China	1	2	5	8	11	12
2008, Xu	China (Shanghai)	-	-	1	2	3	15
2001, Paik	South Korea	-	2	4	7	12	35

* Approximate prevalence of patients with FPHL in each age group.

**Table 2:** Review of association analysis for SNPs with M-AGA in FPHL studies [12,34,36,37,43,44,45,46,47].

Han Chinese *		
Locus	SNP	Association with FPHL
1p36.22	rs12565727	No significant association
2q35	rs10193725	No significant association
	rs7349332	
2q37.3	rs9752491	No significant association
	rs9711321	
	rs12613833	
3q25.1	rs4679955	No significant association
5q33.3	rs929626	No significant association
	rs1081073	
7p21.1	rs2249817	No significant association
	rs12056282	
	rs756853	
	rs13230142	
	rs17350355	
12p12.1	rs7975017	No significant association
	rs9668810	
18q12.3	rs10502861	No significant association
20p11	rs6137444	No significant association
	rs2180439	
	rs1998076	
	rs6113491	
	rs201571	
**UK/Germany ^#^**		
AR/EDA2R	rs4827379	Significant association in a subgroup with an early age of onset
	rs1397631	
	rs2497938	
	rs962458	
	rs6152	
	rs7885198	
	rs5918801	
1p36.22	rs2003046	No significant association
	rs12565727	
	rs11576658	
2q35	rs10193725	No significant association
	rs7349332	
2q37.3	rs9711321	No significant association
	rs11683401	
	rs9287638	
3q25.1	rs7648585	No significant association
	rs4679955	
5q33.3	rs929626	No significant association
	rs1081073	
7p21.1	rs957958	No significant association
	rs2073963	
	rs2073964	
7q11.22	rs6947344	No significant association
	rs6945541	
	rs4718865	
12p12.1	rs9668810	No significant association
	rs7975017	
17q21.31	rs12373124	No significant association
	rs17769552	
	rs17650991	
18q21.1	rs8083006	No significant association
	rs10502861	
	rs12959797	
20p11	rs6137444	No significant association
	rs2180439	
	rs1998076	
	rs201571	
	rs6113491	
CYP19A1	rs16964189	No significant association
	rs4646	
	rs28757184	
	rs2470158	
ESR1	[44]	No significant association
PGR		
SRD5A1		
SRD5A2		
ESR2	rs10137185	Significant association in the overall German sample
MC4R	[47]	No significant association
**Australia ^$^**		
CYP19A1	rs4646	CC genotype is significantly more frequent in some cases
ESR2	rs10137185	Nominal significance
	rs17101774	
	rs2022748	

* reference: [43], ^#^ references: [37,44,45,46,47], ^$^ references: [34,36].

**Table 3:** Summary table of key points for rapid differential diagnosis of common non-scarring alopecia.

	Clinical Presentation	Histopathological Findings	Related Genes
**FPHL**	Widening of the central part line Preservation of the hairline shape Miniaturization of hair follicles Increased hair shedding (not always present)	Increased number of miniaturized hair follicles, a terminal to vellus ratio (T/V ratio) usually less than 2:1 Reduced size of sebaceous glands Decreased anagen to telogen ratio Increased number of follicular stelae Perifollicular inflammation around the upper portion of the hair follicle with or without perifollicular fibrosis	CYP19A1 ESR2
**M-AGA**	Gradual hair thinning in a distinct pattern (typically at the temples and crown) Miniaturization of hair follicles leading to finer, shorter hairs Increased hair shedding, especially during washing or brushing		AR MC4R EDA2R SRD5A1 SRD5A2 [2,47]
**Alopecia areata**	Patchy hair loss in round or oval-shaped areas Hair loss can progress to involve the entire scalp (alopecia totalis) or the entire body (alopecia universalis)	“Swarm of Bees” pattern Peribulbar lymphocytic inflammation Catagen/telogen arrest Follicular miniaturization Exclamation mark hairs Dystrophic anagen hairs	NOTCH4 C6orf10 BTNL2 HLA-DRA HLA-A IL-2 IL2RA STX17 TNXB [71,72]
**Chronic Telogen effluvium**	Excessive shedding of telogen (resting) hairs for more than six months Generalized thinning of hair all over the scalp without visible bald patches or specific pattern Hair regrowth usually occurs once the underlying cause is addressed	Increased number of telogen hairs No peribulbar inflammation Preserved follicular structure Absence of miniaturization	Cdx1 VDR Taq1 VDR [73]

## Data Availability

No new data were created or analyzed in this study. Data sharing is not applicable to this article.

## References

[1] Bertoli M.J., Sadoughifar R., Schwartz R.A., Lotti T.M., Janniger C.K. (2020). Female pattern hair loss: A comprehensive review. Dermatology.

[2] Lolli F., Pallotti F., Rossi A., Fortuna M.C., Caro G., Lenzi A., Sansone A., Lombardo F. (2017). Androgenetic alopecia: A review. Endocrine.

[3] Ludwig E. (1977). Classification of the types of androgenetic alopecia (common baldness) occurring in the female sex. Br. J. Dermatol..

[4] Olsen E.A. (1999). The midline part: An important physical clue to the clinical diagnosis of androgenetic alopecia in women. J. Am. Acad. Dermatol..

[5] Biondo S., Goble D., Sinclair R. (2004). Women who present with female pattern hair loss tend to underestimate the severity of their hair loss. Br. J. Dermatol..

[6] Starace M., Orlando G., Alessandrini A., Piraccini B.M. (2020). Female Androgenetic Alopecia: An Update on Diagnosis and Management. Am. J. Clin. Dermatol..

[7] Davis D.S., Callender V.D. (2018). Review of quality of life studies in women with alopecia. Int. J. Womens Dermatol..

[8] Schmidt S., Fischer T.W., Chren M.M., Strauss B.M., Elsner P. (2001). Strategies of coping and quality of life in women with alopecia. Br. J. Dermatol..

[9] Chaikittisilpa S., Rattanasirisin N., Panchaprateep R., Orprayoon N., Phutrakul P., Suwan A., Jaisamrarn U. (2022). Prevalence of female pattern hair loss in postmenopausal women: A cross-sectional study. Menopause.

[10] Youssef S.M.E., Atallah R.B., Zaky M.S., Eldeek B.S., Elsaie M.L. (2022). Urban-rural differences in the prevalence of female pattern hair loss among secondary school girls: A cross-sectional study. J. Cosmet. Dermatol..

[11] Ramos P.M., Miot H.A. (2015). Female Pattern Hair Loss: A clinical and pathophysiological review. Bras. Dermatol..

[12] Redler S., Messenger A.G., Betz R.C. (2017). Genetics and other factors in the aetiology of female pattern hair loss. Exp. Dermatol..

[13] Knopp E. (2015). The scalp biopsy for hair loss and its interpretation. Semin. Cutan. Med. Surg..

[14] Ieremia E., Stefanato C.M. (2023). The role of hair follicle counts and ratios in the histopathological assessment of androgenic alopecia, alopecia areata and telogen effluvium: Does counting count?. Hum. Pathol..

[15] Peyravian N., Deo S., Daunert S., Jimenez J.J. (2020). The Inflammatory Aspect of Male and Female Pattern Hair Loss. J. Inflamm. Res..

[16] Heilmann-Heimbach S., Hochfeld L.M., Paus R., Nöthen M.M. (2016). Hunting the genes in male-pattern alopecia: How important are they, how close are we and what will they tell us?. Exp. Dermatol..

[17] Birch M.P., Messenger A.G. (2001). Genetic factors predispose to balding and non-balding in men. Eur. J. Dermatol..

[18] Ohn J., Son H.Y., Yu D.A., Kim M.S., Kwon S., Park W.S., Kim J.I., Kwon O. (2022). Early onset female pattern hair loss: A case-control study for analyzing clinical features and genetic variants. J. Dermatol. Sci..

[19] Piccini I., Sousa M., Altendorf S., Jimenez F., Rossi A., Funk W., Bíró T., Paus R., Seibel J., Jakobs M. (2022). Intermediate Hair Follicles from Patients with Female Pattern Hair Loss Are Associated with Nutrient Insufficiency and a Quiescent Metabolic Phenotype. Nutrients.

[20] Fawzi M.M., Mahmoud S.B., Ahmed S.F., Shaker O.G. (2016). Assessment of vitamin D receptors in alopecia areata and androgenetic alopecia. J. Cosmet. Dermatol..

[21] Seleit I., Bakry O.A., Badr E., Mabrouk M. (2020). Vitamin D Receptor Gene Polymorphisms Taq-1 and Cdx-1 in Female Pattern Hair Loss. Indian J. Dermatol..

[22] Banihashemi M., Nahidi Y., Meibodi N.T., Jarahi L., Dolatkhah M. (2016). Serum Vitamin D3 Level in Patients with Female Pattern Hair Loss. Int. J. Trichol..

[23] Jiang V.S., Hawkins S.D., McMichael A. (2022). Female pattern hair loss and polycystic ovarian syndrome: More than just hirsutism. Curr. Opin. Endocrinol. Diabetes Obes..

[24] Tu Y.A., Lin S.J., Chen P.L., Chou C.H., Huang C.C., Ho H.N., Chen M.J. (2019). HSD3B1 gene polymorphism and female pattern hair loss in women with polycystic ovary syndrome. J. Med. Assoc..

[25] Mahmoud E.A., Elgarhy L.H., Hasby E.A., Mohammad L. (2019). Dickkopf-1 Expression in Androgenetic Alopecia and Alopecia Areata in Male Patients. Am. J. Dermatol..

[26] Kwack M.H., Jun M.S., Sung Y.K., Kim J.C., Kim M.K. (2020). Ectodysplasin-A2 induces dickkopf 1 expression in human balding dermal papilla cells overexpressing the ectodysplasin A2 receptor. Biochem. Biophys. Res. Commun..

[27] Hashimoto M., Kawai Y., Masutani T., Tanaka K., Ito K., Iddamalgoda A. (2022). Effects of watercress extract fraction on R-spondin 1-mediated growth of human hair. Int. J. Cosmet. Sci..

[28] Yoshida Y., Takahashi M., Yamanishi H., Nakazawa Y., Kishimoto J., Ohyama M. (2022). Changes in the Expression of Smooth Muscle Cell-Related Genes in Human Dermal Sheath Cup Cells Associated with the Treatment Outcome of Autologous Cell-Based Therapy for Male and Female Pattern Hair Loss. Int. J. Mol. Sci..

[29] Tsuboi R., Niiyama S., Irisawa R., Harada K., Nakazawa Y., Kishimoto J. (2020). Autologous cell-based therapy for male and female pattern hair loss using dermal sheath cup cells: A randomized placebo-controlled double-blinded dose-finding clinical study. J. Am. Acad. Dermatol..

[30] Martinez-Jacobo L., Villarreal-Villarreal C.D., Ortiz-López R., Ocampo-Candiani J., Rojas-Martínez A. (2018). Genetic and molecular aspects of androgenetic alopecia. Indian J. Dermatol. Venereol. Leprol..

[31] Bhat Y.J., Saqib N.U., Latif I., Hassan I. (2020). Female Pattern Hair Loss-An Update. Indian Dermatol. Online J..

[32] Sánchez P., Serrano-Falcón C., Torres J.M., Serrano S., Ortega E. (2018). 5α-Reductase isozymes and aromatase mRNA levels in plucked hair from young women with female pattern hair loss. Arch. Dermatol. Res..

[33] Łukasik A., Kozicka K., Pisarek A., Wojas-Pelc A. (2022). The role of CYP19A1 and ESR2 gene polymorphisms in female androgenetic alopecia in the Polish population. Postep. Dermatol. Alergol..

[34] Yip L., Zaloumis S., Irwin D., Severi G., Hopper J., Giles G., Harrap S., Sinclair R., Ellis J. (2012). Association analysis of oestrogen receptor beta gene (ESR2) polymorphisms with female pattern hair loss. Br. J. Dermatol..

[35] Redler S., Birch P., Drichel D., Hofmann P., Dobson K., Böhmer A.C., Becker J., Giehl K.A., Tazi-Ahnini R., Kruse R. (2014). The oestrogen receptor 2 (ESR2) gene in female-pattern hair loss: Replication of association with rs10137185 in German patients. Br. J. Dermatol..

[36] Yip L., Zaloumis S., Irwin D., Severi G., Hopper J., Giles G., Harrap S., Sinclair R., Ellis J. (2009). Gene-wide association study between the aromatase gene (CYP19A1) and female pattern hair loss. Br. J. Dermatol..

[37] Redler S., Birch M.P., Drichel D., Dobson K., Brockschmidt F.F., Tazi-Ahnini R., Giehl K.A., Kluck N., Kruse R., Lutz G. (2011). Investigation of variants of the aromatase gene (CYP19A1) in female pattern hair loss. Br. J. Dermatol..

[38] Rui W., Sheng Y., Hu R., Miao Y., Han Y., Guo X., Qi S., Xu F., Xu J., Yang Q. (2015). Association of Single Nucleotide Polymorphisms in the CYP19A1 Gene with Female Pattern Hair Loss in a Chinese Population. Dermatology.

[39] Endo Y., Takahashi M., Obayashi Y., Serizawa T., Murakoshi M., Ohyama M. (2017). The ovariectomized mouse simulates the pathophysiology of postmenopausal female pattern hair loss. J. Dermatol. Sci..

[40] Endo Y., Obayashi Y., Ono T., Serizawa T., Murakoshi M., Ohyama M. (2018). Reversal of the hair loss phenotype by modulating the estradiol-ANGPT2 axis in the mouse model of female pattern hair loss. J. Dermatol. Sci..

[41] Pirastu N., Joshi P.K., de Vries P.S., Cornelis M.C., McKeigue P.M., Keum N., Franceschini N., Colombo M., Giovannucci E.L., Spiliopoulou A. (2017). GWAS for male-pattern baldness identifies 71 susceptibility loci explaining 38% of the risk. Nat. Commun..

[42] Kanti V., Messenger A., Dobos G., Reygagne P., Finner A., Blumeyer A., Trakatelli M., Tosti A., Del Marmol V., Piraccini B.M. (2018). Evidence-based (S3) guideline for the treatment of androgenetic alopecia in women and in men - short version. J. Eur. Acad. Dermatol. Venereol..

[43] Liang B., Ding Y., Zhou Y., Yang C., Cheng Z. (2021). Evaluation of Susceptibility Genes/Loci Associated with Male Androgenetic Alopecia (MAGA) for Female-Pattern Hair Loss in a Chinese Han Population and a Brief Literature Review. Med. Sci. Monit..

[44] Redler S., Brockschmidt F.F., Tazi-Ahnini R., Drichel D., Birch M.P., Dobson K., Giehl K.A., Herms S., Refke M., Kluck N. (2012). Investigation of the male pattern baldness major genetic susceptibility loci AR/EDA2R and 20p11 in female pattern hair loss. Br. J. Dermatol..

[45] Redler S., Dobson K., Drichel D., Heilmann S., Wolf S., Brockschmidt F.F., Tazi-Ahnini R., Birch P., Teßmann P., Giehl K.A. (2013). Investigation of six novel susceptibility loci for male androgenetic alopecia in women with female pattern hair loss. J. Dermatol. Sci..

[46] Nuwaihyd R., Redler S., Heilmann S., Drichel D., Wolf S., Birch P., Dobson K., Lutz G., Giehl K.A., Kruse R. (2014). Investigation of four novel male androgenetic alopecia susceptibility loci: No association with female pattern hair loss. Arch. Dermatol. Res..

[47] Mahmoudi H., Redler S., Birch P., Drichel D., Dobson K., Tazi-Ahnini R., Teßmann P., Giehl K.A., Kruse R., Lutz G. (2013). Selected variants of the melanocortin 4 receptor gene (MC4R) do not confer susceptibility to female pattern hair loss. Arch. Dermatol. Res..

[48] Liu Q., Tang Y., Huang Y., Wang J., Yang K., Zhang Y., Pu W., Liu J., Shi X., Ma Y. (2022). Insights into male androgenetic alopecia using comparative transcriptome profiling: Hypoxia-inducible factor-1 and Wnt/β-catenin signalling pathways. Br. J. Dermatol..

[49] van Zuuren E.J., Fedorowicz Z., Schoones J. (2016). Interventions for female pattern hair loss. Cochrane Database Syst. Rev..

[50] Valente Duarte de Sousa I.C., Tosti A. (2013). New investigational drugs for androgenetic alopecia. Expert Opin. Investig. Drugs.

[51] Ocampo-Garza J., Griggs J., Tosti A. (2019). New drugs under investigation for the treatment of alopecias. Expert Opin. Investig. Drugs.

[52] Paichitrojjana A., Paichitrojjana A. (2022). Platelet Rich Plasma and Its Use in Hair Regrowth: A Review. Drug Des. Devel..

[53] Torabi P., Behrangi E., Goodarzi A., Rohaninasab M. (2020). A systematic review of the effect of platelet-rich plasma on androgenetic alopecia of women. Dermatology.

[54] Egger A., Resnik S.R., Aickara D., Maranda E., Kaiser M., Wikramanayake T.C., Jimenez J.J. (2020). Examining the Safety and Efficacy of Low-Level Laser Therapy for Male and Female Pattern Hair Loss: A Review of the Literature. Skin Appendage Disord..

[55] Iamsumang W., Leerunyakul K., Suchonwanit P. (2020). Finasteride and Its Potential for the Treatment of Female Pattern Hair Loss: Evidence to Date. Drug Des. Devel..

[56] Gupta A.K., Talukder M. (2022). Topical finasteride for male and female pattern hair loss: Is it a safe and effective alternative?. J. Cosmet. Dermatol..

[57] Suchonwanit P., Iamsumang W., Leerunyakul K. (2022). Topical finasteride for the treatment of male androgenetic alopecia and female pattern hair loss: A review of the current literature. J. Dermatol. Treat..

[58] Olszewska M., Rudnicka L. (2005). Effective treatment of female androgenic alopecia with dutasteride. J. Drugs Dermatol..

[59] Burns L.J., De Souza B., Flynn E., Hagigeorges D., Senna M.M. (2020). Spironolactone for treatment of female pattern hair loss. J. Am. Acad. Dermatol..

[60] Ramos P.M., Sinclair R.D., Kasprzak M., Miot H.A. (2020). Minoxidil 1 mg oral versus minoxidil 5% topical solution for the treatment of female-pattern hair loss: A randomized clinical trial. J. Am. Acad. Dermatol..

[61] Nascimento E.S.M., Ramos P.M., Silva M.R., Nascimento E.S.R., Barbosa Raposo N.R. (2022). Randomized clinical trial of low-dose oral minoxidil for the treatment of female pattern hair loss: 0.25 mg versus 1 mg. J. Am. Acad. Dermatol..

[62] Saini K., Mysore V. (2021). Role of vitamin D in hair loss: A short review. J. Cosmet. Dermatol..

[63] Zempleni J., Hassan Y.I., Wijeratne S.S. (2008). Biotin and biotinidase deficiency. Expert Rev. Endocrinol. Metab..

[64] Fischer T.W., Hipler U.C., Elsner P. (2007). Effect of caffeine and testosterone on the proliferation of human hair follicles in vitro. Int. J. Dermatol..

[65] Fischer T.W., Slominski A., Tobin D.J., Paus R. (2008). Melatonin and the hair follicle. J. Pineal. Res..

[66] Plonka P.M., Handjiski B., Popik M., Michalczyk D., Paus R. (2005). Zinc as an ambivalent but potent modulator of murine hair growth in vivo- preliminary observations. Exp. Dermatol..

[67] Ibrahim I.M., Hasan M.S., Elsabaa K.I., Elsaie M.L. (2021). Pumpkin seed oil vs. minoxidil 5% topical foam for the treatment of female pattern hair loss: A randomized comparative trial. J. Cosmet. Dermatol..

[68] Campiche R., Le Riche A., Edelkamp J., Botello A.F., Martin E., Gempeler M., Bertolini M. (2022). An extract of Leontopodium alpinum inhibits catagen development ex vivo and increases hair density in vivo. Int. J. Cosmet. Sci..

[69] Nakamura T., Yamamura H., Park K., Pereira C., Uchida Y., Horie N., Kim M., Itami S. (2018). Naturally Occurring Hair Growth Peptide: Water-Soluble Chicken Egg Yolk Peptides Stimulate Hair Growth Through Induction of Vascular Endothelial Growth Factor Production. J. Med. Food.

[70] Moon I.J., Yoon H.K., Kim D., Choi M.E., Han S.H., Park J.H., Hong S.W., Cho H., Lee D.K., Won C.H. (2023). Efficacy of Asymmetric siRNA Targeting Androgen Receptors for the Treatment of Androgenetic Alopecia. Mol. Pharm..

[71] Simakou T., Butcher J.P., Reid S., Henriquez F.L. (2019). Alopecia areata: A multifactorial autoimmune condition. J. Autoimmun..

[72] Tafazzoli A., Forstner A.J., Broadley D., Hofmann A., Redler S., Petukhova L., Giehl K.A., Kruse R., Blaumeiser B., Böhm M. (2018). Genome-Wide MicroRNA Analysis Implicates miR-30b/d in the Etiology of Alopecia Areata. J. Invest. Dermatol..

[73] Seleit I., Bakry O.A., Badr E., Hassan E.H. (2019). Vitamin D Receptor Gene Polymorphism In Chronic Telogen Effluvium; A Case-Control Study. Clin. Cosmet. Investig. Dermatol..

